# A Multiplexed Quantitative Proteomics Approach to the Human Plasma Protein Signature

**DOI:** 10.3390/biomedicines12092118

**Published:** 2024-09-18

**Authors:** Estefanía Núñez, María Gómez-Serrano, Enrique Calvo, Elena Bonzon-Kulichenko, Marco Trevisan-Herraz, José Manuel Rodríguez, Fernando García-Marqués, Ricardo Magni, Enrique Lara-Pezzi, José Luis Martín-Ventura, Emilio Camafeita, Jesús Vázquez

**Affiliations:** 1 Centro Nacional de Investigaciones Cardiovasculares Carlos III, 28029 Madrid, Spain; enunez@cnic.es (E.N.); ecalvo@cnic.es (E.C.); elena.bonzon@uclm.es (E.B.-K.); jmrodriguezc@cnic.es (J.M.R.); rmagni@gmail.com (R.M.); elara@cnic.es (E.L.-P.); 2 CIBER de Enfermedades Cardiovasculares (CIBERCV), 28029 Madrid, Spain; jlmartin@fjd.es; 3 Institute for Tumor Immunology, Center for Tumor Biology and Immunology (ZTI), Philipps University, 35043 Marburg, Germany; maria.gomezserrano@imt.uni-marburg.de; 4 International Center for Life, Newcastle University, Newcastle upon Tyne NE1 4EP, UK; marco.trevisan@newcastle.ac.uk; 5 Canary Center for Cancer Early Detection, Stanford, CA 94304, USA; fjgarcia@stanford.edu; 6 IIS-Fundación Jiménez-Díaz, 28015 Madrid, Spain

**Keywords:** LC-MS/MS, human plasma, plasma proteomics, clinical proteomics, atherosclerosis, personalized medicine

## Abstract

Despite the plasma proteome being able to provide a unique insight into the health and disease status of individuals, holding singular promise as a source of protein biomarkers that could be pivotal in the context of personalized medicine, only around 100 proteins covering a few human conditions have been approved as biomarkers by the US Food and Drug Administration (FDA) so far. Mass spectrometry (MS) currently has enormous potential for high-throughput analysis in clinical research; however, plasma proteomics remains challenging mainly due to the wide dynamic range of plasma protein abundances and the time-consuming procedures required. We applied a new MS-based multiplexed proteomics workflow to quantitate proteins, encompassing 67 FDA-approved biomarkers, in >1300 human plasma samples from a clinical cohort. Our results indicate that this workflow is suitable for large-scale clinical studies, showing good accuracy and reproducibility (coefficient of variation (CV) < 20 for 90% of the proteins). Furthermore, we identified plasma signature proteins (stable in time on an individual basis), stable proteins (exhibiting low biological variability and high temporal stability), and highly variable proteins (with low temporal stability) that can be used for personalized health monitoring and medicine.

## 1. Introduction

Blood is the primary link between different parts of the body and has the potential to expose the health/pathological status of any key organ. Plasma and serum are the predominant clinical specimens available for routine molecular analysis, and among the molecules present in blood, proteins have the greatest clinical significance. Plasma is estimated to contain more than 20,000 different proteins (based on the number of human protein-coding genes) with concentrations spanning 10–12 orders of magnitude. Thus, albumin constitutes 60% of the total plasma proteome, with a concentration of 50 mg/ml, whereas the concentration of interleukin-6 is 4.2 pg/mL [1,2]. In addition, alternative splicing and post-translational modifications (PTMs) lead to millions of different proteoforms [3], not to mention the underexplored peptidome [4] and the non-canonical proteome [5]. Thousands of proteins have been measured in plasma or serum using either MS-based proteomics, affinity-based proteomics, or combinations thereof, but to date, only around 100 plasma protein biomarkers have been approved by the FDA [6].

MS is the most commonly used biomolecular detection technique in proteomics, allowing high-throughput analysis of proteins in biological samples with almost complete coverage [7,8]. MS is the technique of choice for the analysis of human samples and body fluids, as it can yield specific quantitative information on every proteome component in an unbiased way and therefore contribute to deciphering the molecular basis of human diseases. However, MS-based analysis of human plasma samples is constrained by the above-mentioned large dynamic range of protein concentrations and complexity. To overcome this drawback and achieve deep and extensive proteome coverage upon liquid chromatography–tandem MS (LC-MS/MS) analysis, sample depletion from highly abundant proteins (i.e., those accounting for >99% of the total protein mass) and extensive peptide-level fractionation via offline separation techniques, such as affinity enrichment [9,10], reversed-phase chromatography at basic pH, and strong cation exchange chromatography, have been used [11,12,13]. However, immunodepletion can not only lead to sample loss and diminished sample-to-sample reproducibility but also cause analytical bias due to the removal of lower abundance proteins carried over by such species as albumin or unspecifically bound to the antibodies used [14,15]; moreover, extensive fractionation at the peptide level is time-consuming and therefore not feasible for large sample sizes [16]. Some of these drawbacks can be circumvented by using multiplexed analysis based on isobaric labeling techniques in combination with peptide-level fractionation to perform large-scale studies with suitable depth in a reasonable time.

Recent advances in MS-based proteomics have led to noticeable improvements in sensitivity, analytical dynamic range, speed, and robustness that hold enormous potential for large-scale plasma proteomics in clinical research. Label-free approaches are more challenging to implement when using very long gradients and peptide fractionation (as required to dig deeper into the plasma proteome) because of the difficulty to maintain constant chromatographic and mass spectrometric performance, two parameters that are essential for accurate quantification, across hundreds or thousands of individual LC-MS/MS runs. On the other hand, isobaric labeling allows routine analysis of large numbers of samples via LC-MS/MS without demanding chromatographic stability over extended time ranges. 

Analysis of >1000 samples from a clinical research cohort of subjects with acceptable throughput and analytical depth using MS-based shotgun proteomics is a challenge. So far, most non-targeted plasma proteomics studies have been confined to the analysis of <100 samples, which offers limited power for biomarker discovery, and only a few works have analyzed >500 human plasma samples with significant proteome coverage. Bruderer et al. [17] described the analysis of 1508 plasma samples at 31 samples/day with a depth of 565 proteins per analysis using capillary-flow data-independent acquisition; however, quality control samples were required to monitor LC-MS/MS performance. Cominetti et al. [18] resorted to multiplexed labeling with tandem mass tags (TMT) to quantitate 1000 samples at 50 samples/day, but only 190 proteins were identified per analysis. More recently, Niu et al. [19], using a data-independent acquisition approach, reported the analysis of 659 plasma samples at 60 samples/day with a depth of ca. 300 proteins per analysis. 

We have recently developed an MS-based proteomics workflow for the high-throughput analysis of human plasma samples based on isobaric labeling of peptides for multiplexed quantification, followed by automated peptide and protein quantification using a robust statistical model previously developed in our laboratory [20,21,22,23,24,25]. This workflow has been used for the analysis of the human plasma proteome in several works [26,27,28,29,30,31,32,33]. In this study, we evaluate in detail the performance and the statistical accuracy of our plasma proteomics workflow and study the evolution of plasma protein abundance over time using previously published data from over 1300 human plasma samples pertaining to the PESA and AWHS cohorts.

## 2. Materials and Methods

### 2.1. Plasma Samples

Plasma samples were collected from the PESA study cohort [34] at baseline (V1, 444 samples) and at the 3-year follow-up visit (V2, 444 samples), as well as from the AWHS cohort [35,36] (350 samples). PESA is a prospective cohort study with asymptomatic employees (age: 40–54 years) of the Santander Bank (Madrid, Spain). AWHS is a prospective longitudinal cohort study of middle-aged workers free of clinical cardiovascular disease (CVD) from the General Motors Spain automobile assembly plant (Zaragoza, Spain).

### 2.2. Plasma Depletion

Immunodepletion of the 14 most abundant plasma proteins was carried out with the Multiple Affinity Removal Column Human 14 (4.6 × 100 mm, Agilent, Santa Clara, CA, USA) coupled with a 1290 Infinity II liquid chromatography system (Agilent).

### 2.3. Protein Digestion

A volume of 5 µl of plasma from each individual (corresponding to 300 µg of protein, as measured by NanoDrop 1000, Thermo Fisher Scientific, Waltham, MA, USA) was mixed with 5 µl of a buffer containing 50 mM Tris, 2% sodium dodecyl sulfate, and 100 mM dithiothreitol and boiled for 5 min. On-filter protein digestion was performed using Nanosep Centrifugal Devices with Omega Membrane 10K (Pall, Portsmouth, UK) following the manufacturer’s instructions. Briefly, 320 µl of urea was added to each sample, and the mixture was transferred to the filter, which was then subjected to centrifugation at 14,000× *g* for 10 min. Cysteine residues were alkylated with 50 mM iodoacetamide with 1 h incubation at room temperature in the dark. After two washes with urea followed by two washes with 100 mM ammonium bicarbonate pH 8.8, proteins were digested with trypsin (1:30 *w*/*w* trypsin: protein, Promega, Madison, WI, USA) overnight at 37 °C. The resulting peptides were eluted with ammonium bicarbonate and NaCl, and the resulting peptide solution was acidified with 25% trifluoroacetic acid (TFA; final concentration, 1%; Merck, Darmstadt, Germany) and desalted with Oasis cartridges (Waters, Milford, MA, USA) following the manufacturer’s instructions. Finally, the eluted peptides were vacuum-dried and stored at −20 °C until further use.

On-plate protein digestion was performed as described above using 96-well plates (AcroPrep 96-well Filter Plates, Pall) coupled to a vacuum manifold (Pall), after which the resulting peptides were desalted in Oasis HLB 96-well plates (Waters). Finally, the peptide solutions were vacuum-dried and stored at −20 °C until further use. 

### 2.4. Isobaric Labeling

Peptide samples were taken up in 100 mM triethylammonium bicarbonate, and the peptide concentration was measured using a DirectDetect infrared spectrometer (Merck). Then, the peptides were subjected to multiplexed isobaric labeling with TMT using the TMT 10plex Isobaric Label Reagent Set (Thermo Fisher Scientific) following the manufacturer’s instructions. A total of 156 TMT experiments were performed, processed in 15-TMT batches. Each TMT experiment comprised eight individuals, with two TMT channels reserved for a reference internal standard sample that was prepared as follows: After protein digestion, equal peptide amounts from half of the samples included in each of the 15-TMT batches were pooled; then, for each TMT experiment, a 50 µg aliquot was labeled with TMT reagent 126, and another 50 µg aliquot was labeled with TMT reagent 131; afterward, these two labeled samples were pooled (to compensate for potential different labeling efficiencies of the two TMT reagents used) and used to spike the eight pooled samples comprising each TMT experiment with the same amount of internal standard sample. The resulting labeled peptide mixtures were acidified with 25% TFA (1% final concentration) and desalted with Oasis cartridges (Waters). Finally, 1/10 and 9/10 aliquots of the eluted peptides were vacuum-dried and stored at −20 °C for later MS analysis and peptide-level fractionation, respectively.

### 2.5. Fractionation of Peptide Samples

The 9/10 aliquot was taken up in 0.1% TFA and separated into five fractions using the High pH Reversed-Phase Peptide Fractionation Kit (Thermo Fisher Scientific) according to the manufacturer’s instructions. Briefly, the cartridges were washed with 50% and 100% acetonitrile (ACN) and equilibrated with 0.1% TFA. Then, the labeled peptide samples were loaded into the cartridges and the peptides eluted in five different fractions with increasing ACN concentration: 12.5% ACN, 15% ACN, 17.5% ACN, 20% ACN, and 50% ACN. The resulting labeled peptide fractions were vacuum-dried and stored at −20 °C for further MS analysis.

### 2.6. LC-MS/MS Analysis

The labeled peptide samples were taken up in 0.1% formic acid (FA) and subjected to LC-MS/MS analysis using an EASY-nLC 1200 liquid chromatography system (Thermo Fisher Scientific) coupled with an Orbitrap Fusion mass spectrometer (Thermo Fisher Scientific) in the case of PESA V1 plasma samples or an Ultimate 3000 HPLC system (Thermo Fisher Scientific) coupled with a Q Exactive HF mass spectrometer (Thermo Fisher Scientific) in the case of PESA V2 and AWHS plasma samples. C18-based reversed-phase separation was carried out using a PepMap 100 C18 trapping column (Thermo Fisher Scientific) and an EASY-Spray 50 cm analytical column (Thermo Fisher Scientific). Peptides were loaded in buffer A (0.1% (*v*/*v*) FA in water) and eluted with an ACN gradient consisting of 0–21% buffer B (100% ACN, 0.1% (*v*/*v*) FA) for 300 min and 21–90% B for 5 min at a flow rate of 200 nl/min. Mass spectra were acquired in a data-dependent manner, with an automatic switch between MS and MS/MS using a top-speed method with the Fusion mass spectrometer and a top-15 method with the Q Exactive HF instrument. MS spectra were acquired in the Orbitrap analyzer in the 400–1500 *m*/*z* range with 70,000 resolution. Higher energy collisional dissociation was performed with a normalized collision energy value of 30, and MS/MS spectra were acquired with 60,000 resolution in the Orbitrap.

### 2.7. Protein Identification

For peptide identification, MS/MS spectra were searched with the SEQUEST HT algorithm implemented in Proteome Discoverer 2.1 (Thermo Scientific) against a concatenated target-decoy database comprising human protein sequences (UniProtKB/Swiss-Prot 2014_07 release) with the following parameters: trypsin digestion with up to 2 missed cleavages; Cys carbamidomethylation (57.021464 Da) and TMT labeling (229.162932 Da) at peptide N-terminus and Lys residues were set as fixed modifications; Met oxidation (15.994915) was allowed as a dynamic modification; and precursor and fragment mass tolerance were set to 800 ppm and 0.02 Da, respectively. The false discovery rate (FDR) for peptide identification was calculated based on the refined method with 15 ppm precursor mass tolerance postfiltering [20,25]. A 1% FDR threshold was considered for peptide identification, and peptides were assigned to the best protein proposed by Proteome Discoverer.

### 2.8. Protein Quantification and Statistical Analysis

Protein quantification and statistical and systems biology analysis were performed based on the quantitative information extracted from the MS/MS spectra of the TMT-labeled peptides using the iSanXoT package [37], based on the models and algorithms previously developed in our laboratory [21,22,24,38]. For peptide quantification, the raw quantitative data were used as inputs to the weighted spectrum, peptide, and protein (WSPP) model to compute the log2 ratio of each individual with respect to the average value of the two internal standard samples. Comparative analysis was carried out by estimating the contribution of spectrum, peptide, and protein variance to the total technical variance; thus, relative protein abundance was measured by Xq, the log2 ratio at the protein level, and by Zq, the log2 ratio at the protein level expressed in units of standard deviation, based on the corresponding variance estimation. The model also enabled the estimation of statistical weight, Wq, for each protein, defined as the inverse of its variance.

To calculate CV, we estimated absolute protein abundances by summing up the reporter intensities of all scans belonging to a given protein and calculated an average across all the internal standard samples included in the TMT batches. We then multiplied the averaged abundance of each protein by $2^{X_{q}}$. The standard deviation was estimated as $1/\sqrt{W_{q}}$.

### 2.9. Functional Enrichment and Clustering Analyses

Functional enrichment and clustering analyses were performed with String v11.0 [39] and Cytoscape v3.7.2 [40], respectively.

### 2.10. Biochemical Measurements

The plasma levels of immunoglobulin heavy constant alpha 2 (IGHA2) and apolipoprotein (a) (LPA) were measured by immunoturbidimetric assays (LK088.OPT and LK098.OPT, respectively, The Binding Site, London, UK) using the Binding Site Optilite analyzer (The Binding Site) in a blinded manner. The plasma levels of C-reactive protein (CRP) and apolipoprotein B-100 (ApoB) were measured as previously published [34].

## 3. Results

### 3.1. Influence of Plasma Depletion on Protein Quantification

**Figure 1:**
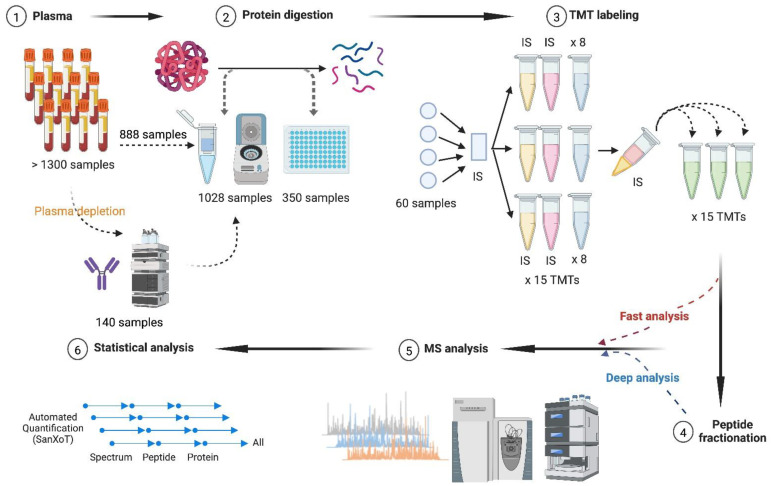
Proteomics workflow for multiplexed analysis of large plasma cohorts. (1) A total of 1378 plasma samples were analyzed, of which 140 samples were subjected to plasma depletion before protein digestion. (2) The same on-filter tryptic digestion procedure was applied using individual centrifugal devices (1028 samples) and the 96-well plate format (350 samples). (3) The resulting peptides were isobarically labeled with TMT for a total of 156 TMT experiments that were processed in 15-TMT batches. Each TMT experiment comprised eight different plasma samples together with the internal standard sample prepared by pooling half of the samples included in each of the 15-TMT batches. (4,5) The labeled peptides were analyzed by LC-MS/MS both before (fast analysis) and after (deep analysis) high-pH reversed-phase fractionation. (6) Protein quantification was accomplished using iSanXoT, an implementation of the WSPP statistical model previously developed in our group. LC-MS/MS, liquid chromatography–tandem mass spectrometry; TMT, tandem mass tags; WSPP, weighted spectrum, peptide, and protein. This figure was created in BioRender (BioRender.com/v03v870).

**Figure 2:**
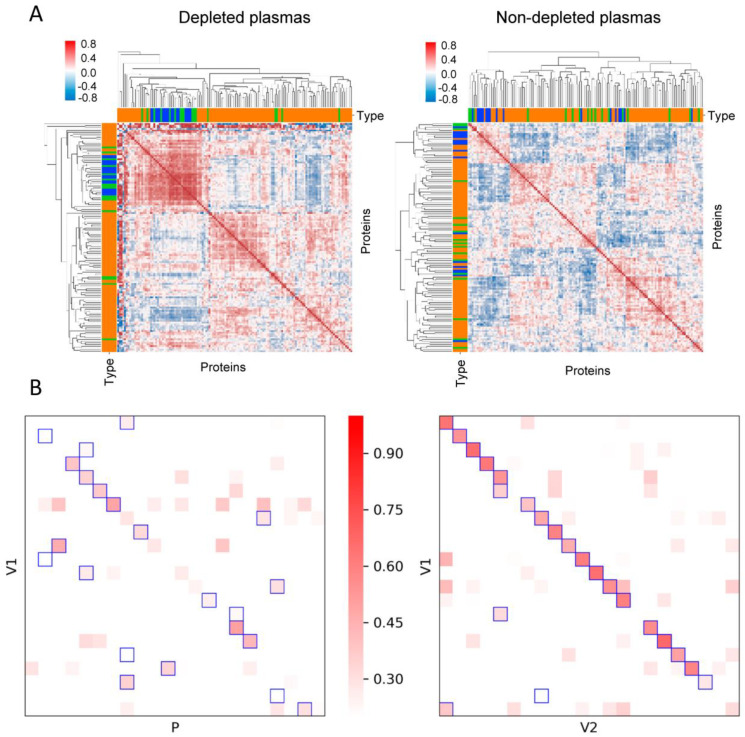
Technical issues in plasma depletion. (**A**) Protein clusters in depleted and non-depleted plasma samples showing that most depletable proteins (green) that could also be quantified in depleted plasmas grouped along with many other proteins (blue) in a single cluster, with other proteins quantified shown in orange (left panel). In the non-depleted plasma samples, depletable proteins distribute among several clusters (right panel). (**B**) Correlation analysis, excluding depletable proteins, between plasma samples from the same individuals with and without depletion (labeled as P and V1, respectively) and between non-depleted plasma samples from the same individuals at baseline (V1) and three years later (V2) (right panel). Squares indicate the best correlation found for individuals from V1.

In this work, we investigated the proteomics results obtained from the analysis of 1008 plasma samples from the PESA study cohort [34], namely 120 samples used as a test cohort, 444 additional baseline samples, and 444 samples at 3-year follow-up visit (Figure 1). Firstly, we analyzed whether depletion was a suitable procedure to increase proteome coverage using the 120-sample test cohort. These plasma samples were depleted from the 14 most abundant proteins prior to filter-aided digestion with trypsin. Then, the resulting peptides were labeled with TMT reagents prior to fractionation using high-pH reversed-phase chromatography. The peptides were analyzed by LC-MS/MS both before (fast analysis) and after fractionation (deep analysis), after which peptide and protein quantification were achieved based on the WSPP model previously developed in our laboratory [21] (Figure 1). Of note, we obtained an average number of 10,957 peptides and 1674 proteins per sample, which suggested that depletion was a promising approach to enhance the depth of analysis of this kind of samples. We then investigated whether depletion produced alterations to protein abundance. We found that after depletion, around 30% of the peptide–spectrum matches (PSMs) belonged to depletable proteins, indicating that these depletable proteins were still present in significant amounts. The clustering analysis revealed that the remaining depletable proteins (highlighted in green in Figure 2A, left panel) gathered at a single main cluster together with many other species (in blue), indicating that all these proteins were affected by the depletion process. In clear contrast, this cluster was not observed when the same 120 plasma samples were processed without prior depletion (see the details below), where both the depletable proteins and the majority of the other proteins found previously clustered did not reveal any evidence of clustering (Figure 2A, right panel). Moreover, the protein abundance (excluding depletable proteins) in each of the 120 depleted samples (P, Figure 2B, left panel) showed low correlation with the corresponding non-depleted samples (V1). In clear contrast, when no depletion was performed, a high intra-individual correlation was found between protein levels at baseline (V1, Figure 2B, right panel) and those from the plasma samples obtained three years later (V2). These results demonstrate that depletion of the most abundant proteins, although successful in improving proteome coverage, introduced a strong variability in the plasma proteome, probably due to the incompleteness of depletion and the existing interactions between depletable and non-depletable proteins. For these reasons, the depletion protocol was judged unsuitable for the analysis of large cohorts.

### 3.2. Performance of the Plasma Proteomics Workflow

The 444 baseline samples (V1) and the 444 samples from the same individuals obtained at the 3-year follow-up visit (V2, see Figure 1) were processed without depletion. Using this approach, we were able to quantify 4604 peptides on average, corresponding to 917 proteins (547 quantified with >1 peptide across at least 60% of the individuals), with the deep analysis, which included fractionation; meanwhile, 3027 peptides, corresponding to 537 proteins (349 detected with >1 peptide across at least 60% of the individuals), were quantitated with the fast analysis.

The fast approach (without fractionation) required 50 min of LC-MS/MS time per sample, allowing the analysis of 29 samples per day. These figures were comparable to those reported with published conventional label-free approaches (21–88 min per sample, depending on the method used, allowing the analysis of 50 and 15 samples per day, respectively). The protein throughput, however, compared favorably (537 versus 241–512 proteins per sample) [41,42,43,44]. In comparison, the deep analysis required 225 min per sample, allowing the analysis of 8 samples per day, but with a depth of more than 900 proteins, a number rarely found in high-throughput plasma proteomics works. Of interest, one representative work in the field reached 965 plasma proteins via label-free analysis using extensive fractionation, but at the cost of considerably longer analysis time (960 min) [45].

### 3.3. Quantification Accuracy of the Plasma Proteomics Workflow

To investigate the quantitative accuracy of the protein quantifications produced using the workflow, we resorted to the WSPP statistical model previously developed in our laboratory, which provides a statistical framework for testing the quality of quantitative experiments and detecting experimental deviations. The WSPP model can separately estimate the variance originated by (i) protein extraction and manipulation (protein-level variance), (ii) protein digestion and subsequent peptide labeling (peptide-level variance), and (iii) extraction of quantitative information from LC-MS/MS data (scan-level variance) [24]. 

**Figure 3:**
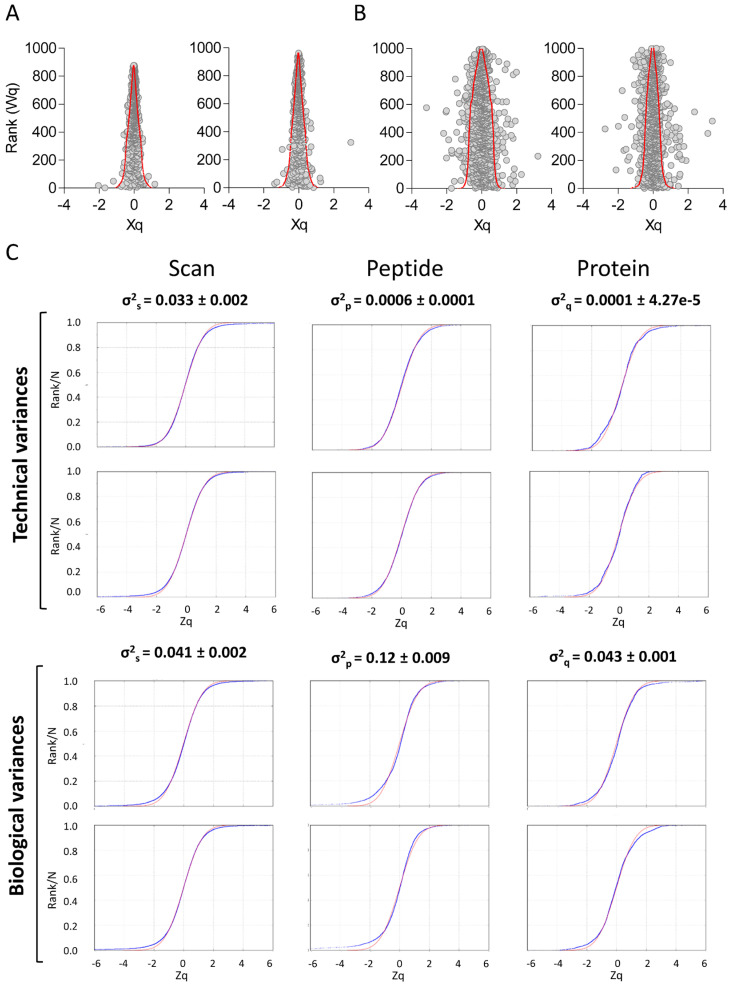
Protein quantification accuracy of the large-scale proteomics workflow. (**A**) Average protein quantification values (grand-mean-corrected) from the same internal standard sample labeled with two different TMT reagents plotted against their ranked statistical weight. Red lines delimit the 95% confidence interval (two standard deviations). (**B**) Average protein quantification values (grand-mean-corrected) from two different biological samples plotted against their ranked statistical weight. Red lines delimit the 95% confidence interval (two standard deviations). (**C**) Technical and biological variances at the scan, peptide, and protein levels. Technical variance (top panel) was estimated using the quantification of the same internal standard labeled with two different TMT tags, whereas biological variance (bottom panel) was estimated by matching the quantification of one individual against the average of the internal standard sample (which was labeled with two different TMT tags). Variance values are expressed as average (from 14 and 56 experiments for technical and biological variance, respectively) ± SEM. SEM, standard error of the mean; TMT, tandem mass tags.

First, to ascertain quantitative reproducibility, we plotted protein quantification values from the same internal standard sample labeled with two different TMT tags against their corresponding statistical weight (i.e., the inverse of variance) (Figure 3A). Protein quantification was generally within the 95% confidence interval, and practically, no significant protein changes were detected, supporting accurate quantification of the internal standard sample. This analysis was also performed with biological samples (Figure 3B). In this case, 5% of the quantified proteins lay outside the confidence limits, which could be accounted for by inter-individual variability of the plasma proteome [46,47,48,49,50,51].

Furthermore, technical variability was assessed by estimating variances at the scan, peptide, and protein levels using the quantification of the same internal standard sample labeled with two different TMT tags (Figure 3C, top panel). As expected for the analysis of technical replicates from identical samples, the variances at the peptide and protein levels were not significantly different from zero, and significant variance, generated by the errors associated with the LC-MS/MS analysis, was only detected at the scan level. To assess biological variability, we analyzed the variances generated from the analysis of separate plasma samples. These were calculated by matching the quantification of one individual against the average of the internal standard sample (which was labeled with two different TMT tags) (Figure 3C, bottom panel). As expected, the variance at the scan level was similar to those of the technical replicates, and non-zero variances were measured at the peptide and protein levels due to biological variability (Figure 3C, bottom panel). These variances were in the ranges previously reported for the analysis of biological samples of very different origin [22]. Of note, the distribution of quantification errors was in all cases in excellent agreement with the theoretical null hypothesis distributions (Figure 3C, red sigmoids), indicating that the statistical model was an accurate approach to analyze the results produced using this workflow. 

**Figure 4:**
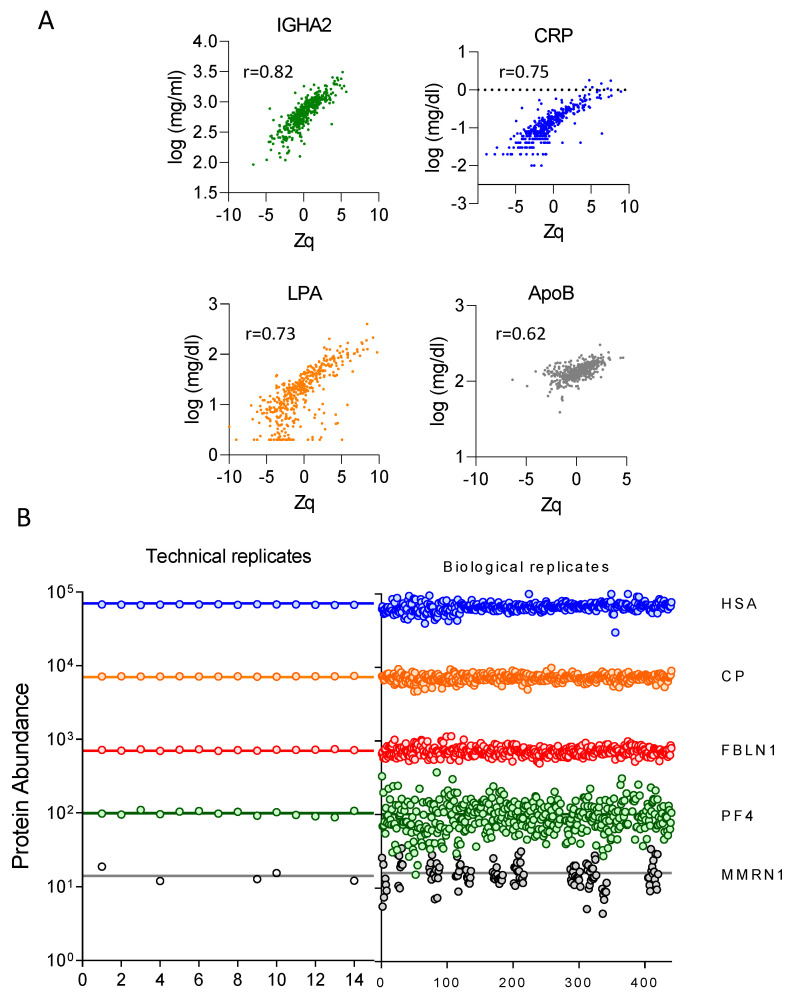
Correlation with biochemical measurements and quantitative reproducibility of the large-scale proteomics workflow. (**A**) Biochemical versus proteomics quantification of immunoglobulin heavy constant alpha 2 (IGHA2), C-reactive protein (CRP), apolipoprotein(a) (LPA), and apolipoprotein B-100 (ApoB), showing a strong correlation (*p*-value < 10^−15^). The Pearson correlation coefficient (r) is indicated. (**B**) Average LFQ intensity showing reproducible quantification across 14 technical replicates (left panel) and 444 biological replicates (right panel) of five selected proteins with plasma concentration spanning five orders of magnitude. Blue line: human albumin, HAS; orange: ceruloplasmin, CP; red: fibulin-1, FBLN1; green: platelet factor 4, PF4; and gray: multimerin-1, MMRN1. LFQ, label-free quantification; Zq, standardized log2 ratio at the protein level.

Finally, prior biochemical measurements of a number of proteins, namely immunoglobulin heavy constant alpha 2 (IGHA2), C-reactive protein (CRP), apolipoprotein(a) (LPA), and apolipoprotein B-100 (APOB), in hundreds of samples [33,34] showed a very strong correlation (*p*-value < 10^−15^) with the quantitative values provided by the workflow, further supporting their high quantitation accuracy (Figure 4A).

### 3.4. Technical and Biological Variability of the Plasma Proteome

To evaluate the technical variability of the plasma proteome, we selected five plasma proteins, namely human serum albumin (HSA), ceruloplasmin (CP), fibulin-1 (FBLN1), platelet factor 4 (PF4), and multimerin-1 (MMRN1), with plasma concentrations spanning five orders of magnitude, and represented their estimated absolute abundances (calculated as the average value of the same internal standard sample labeled with two different TMT tags). The quantification was reproducible across the 14 technical replicates for the five proteins selected (Figure 4B, left). The absolute abundance of these five proteins across 444 individuals distributed among 56 TMT experiments showed the expected biological variability of these samples around stable average values (Figure 4B, right). 

**Figure 5:**
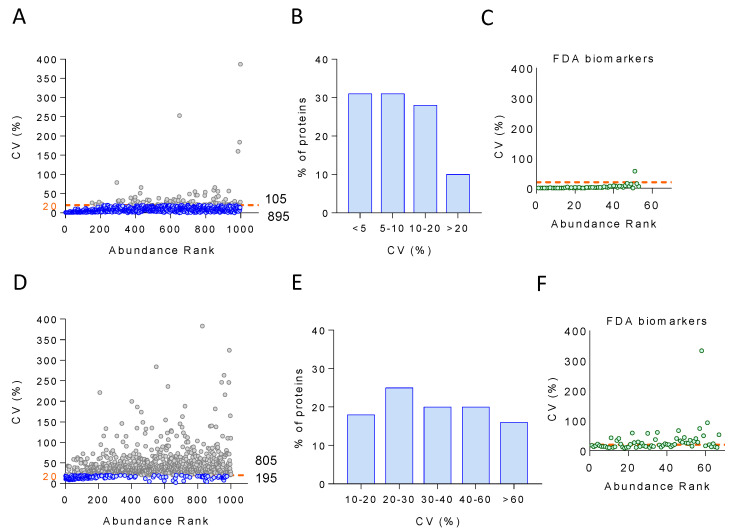
Analytical and biological variability of the large-scale proteomics workflow. (**A**) Analytical CV for the 1000 most abundant proteins quantitated across 14 technical replicates as a function of their ranked abundance. A total of 895 proteins showed CV < 20 (blue dots), and 105 proteins showed CV > 20 (gray dots). (**B**) Analytical CV values distributed as follows: 31% of the proteins had CV < 5; 31% ranged from 5 to 10; 28% ranged from 10 to 20; and 10% showed CV > 20. (**C**) Analytical variability of the FDA-approved biomarkers. The analytical CV of the 67 FDA biomarkers that could be quantitated was plotted as a function of their ranked abundance. Only one of the biomarkers showed CV > 20 (orange dashed line). (**D**) Biological CV for the 1000 most abundant proteins quantitated across 444 individuals as a function of their ranked abundance. A total of 195 proteins showed CV < 20 (blue dots), and 805 proteins showed CV > 20 (gray dots). (**E**) Biological CV values distributed as follows: 18% of the proteins had CV from 10 to 20; 25% ranged from 20 to 30; 20% ranged from 30 to 40; 20% ranged from 40 to 60; and 16% showed CV > 60. (**F**) Biological variability of the FDA biomarkers. The biological CV of the 67 FDA biomarkers that could be quantitated was plotted as a function of their ranked abundance. A total of 33 proteins showed CV > 20 (orange dashed line). CV, coefficient of variation; FDA, US Food and Drug Administration.

To investigate the analytical variability, we plotted CV for the 1000 most abundant proteins quantified across 14 technical replicates against their ranked abundance. The results indicate that 90% of the proteins (895) had CV < 20%, which is the most common cut-off in diagnostic assays [52] (Figure 5A), whereas 31% of the proteins showed CV < 5 (Figure 5B). Moreover, we were able to quantify 67 out of the 109 FDA-approved biomarkers [52], with only one of them, one of the less abundant species, showing a CV > 20 (Figure 5C). Inter-individual (biological) variability was estimated by calculating the CV for the 1000 most abundant proteins quantified across the 444 individuals that were analyzed via LC-MS/MS over four months. In this case, 20% of the proteins (195) showed CV ranging from 10 to 20, and 25% between 20 and 30 (Figure 5D,E). Regarding the FDA-approved biomarkers, half of them showed CV < 20 (Figure 5F).

**Figure 6:**
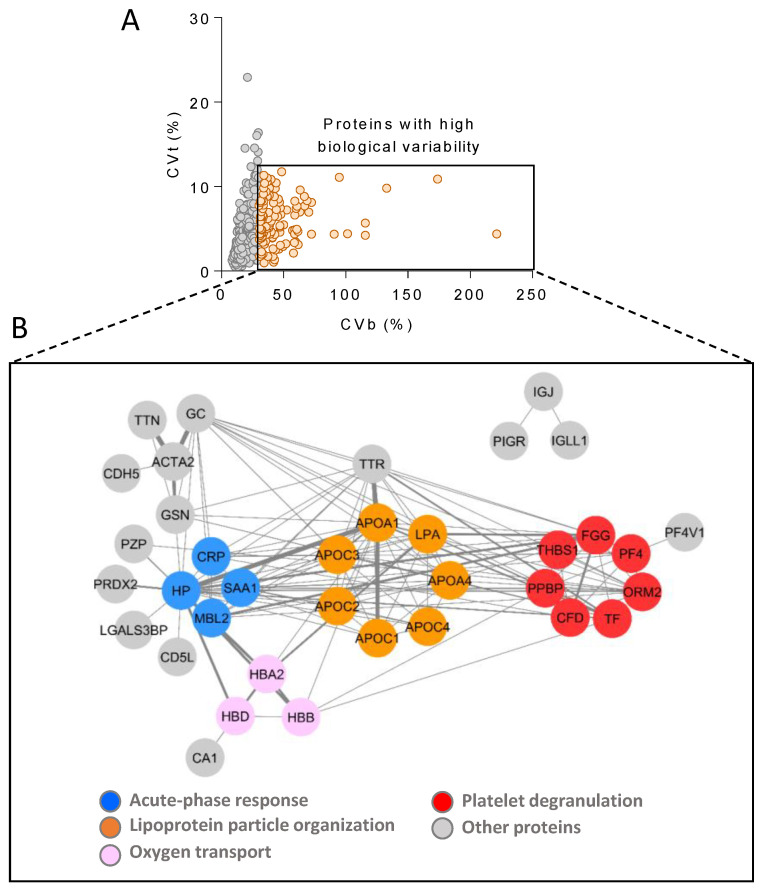
Highly biologically variable proteins. (**A**) Analytical variability (CVt) was plotted versus biological variability (CVb) for proteins quantitated across at least 80% of the individuals at baseline. Proteins with CVt < 12 and CVb > 30 were considered to have high biological variability (orange dots). (**B**) Correlation network obtained with the highly biologically variable proteins. Edge line thickness is proportional to the strength of the association. The following functional categories were found enriched (FDR < 1%): acute-phase response (blue), lipoprotein particle organization (orange), oxygen transport (pink), and platelet degranulation (red). Additional highly biologically variable proteins are highlighted in gray. CVb, biological coefficient of variation; CVt, technical coefficient of variation; FDR, false discovery rate.

Next, we set out to examine in more detail the nature of the proteins showing very high biological variability not due to technical variability, i.e., those quantitated across at least 80% of the individuals with technical CV (CVt) < 12 and biological CV (CVb) > 30 (Figure 6A). The functional enrichment analysis of these proteins revealed acute-phase response, lipoprotein particle organization, oxygen transport, and platelet degranulation as enriched (at 1% FDR) functional categories (Figure 6B).

### 3.5. Long-Term Temporal Stability of the Plasma Proteome 

**Figure 7:**
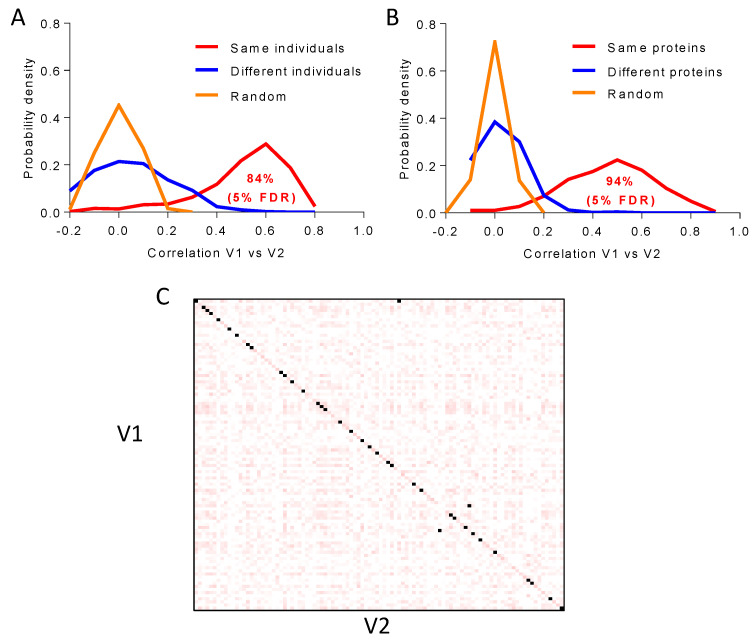
Long-term stability of the plasma proteome. (**A**) Probability distribution of the Pearson correlation coefficient between plasma protein quantitative values at baseline (V1) and three years later (V2), showing statistically significant temporal intra-individual correlation (5% FDR) between protein quantitative values for 84% of the individuals (red line). No correlation was found between different (blue line) or random (orange line) individuals. (**B**) Probability distribution of the Pearson correlation coefficient between the plasma protein quantitative values at baseline (V1) and three years later (V2), showing statistically significant temporal intra-protein correlation (5% FDR) for 94% of the proteins quantitated across individuals (red line). No correlation was found between different (blue line) or random (orange line) proteins. (**C**) The Pearson correlation matrix of plasma proteins quantitated across 440 individuals (224 proteins) in V1 and V2. For most of the subjects (90%), intra-individual correlation was higher than that between proteins from different individuals. FDR, false discovery rate.

Protein quantitative values from the same individual showed a significant correlation (FDR < 5%) between baseline (V1) and three years later (V2) in 84% of the individuals; this correlation was not found when comparing the proteome of different individuals or upon random comparison (Figure 7A). Likewise, for 94% of the proteins, a significant correlation (FDR < 5%) was obtained between the quantification values of the protein across all individuals at baseline and the values of the same protein across the same individuals three years later; once again, this correlation was not found when comparing different proteins or upon random comparison (Figure 7B). We also found that the proteins from 25% of the individuals in V1 did not show a correlation higher than 0.42 with any of the individuals in V1, indicating that the plasma underwent significant technical or biological alterations. In clear contrast, in the remaining 75% of individuals, the correlation matrix built with the plasma proteins quantitated at baseline and three years later showed that, for most (90%) of the individuals, the correlation is higher between proteins from the same individual as compared with correlation across different subjects (diagonal dots in Figure 7C). This is an extremely improbable result (*p*-value < 10^−15^), since only 1–2 individuals are expected to match the same individual by chance alone in a population of ca. 300 samples. These results indicate that the plasma proteome of each individual is highly stable over time and can be recognized as long as three years later in the majority of cases.

### 3.6. Classification of Plasma Proteins According to Temporal Stability and Biological Variability

**Figure 8:**
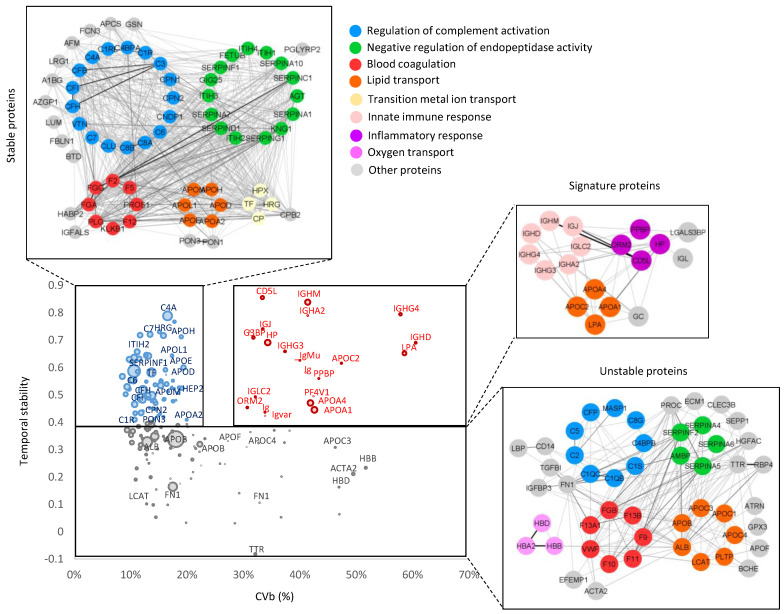
Temporal stability and biological variability of plasma proteins. The Pearson correlation coefficient between protein quantitative values at baseline and three years later (temporal stability) versus CVb. Only proteins with CVt < 12 were considered. Proteins were classified into three groups: stable proteins (Pearson coefficient > 0.4 and CVb < 20), signature proteins (Pearson coefficient > 0.4 and CVb > 30), and unstable proteins (Pearson coefficient < 0.4). The insets show correlation across protein components of the functional categories found enriched (FDR < 1%) in each protein group, with edge line thickness proportional to correlation strength. CVb, biological coefficient of variation; CVt, technical coefficient of variation; FDR, false discovery rate.

To further study the plasma proteome in terms of temporal stability and biological variability, we plotted CVb against the temporal stability of those proteins quantified with a CVt < 12. Following the results in Figure 7C, temporal stability was measured by the Pearson correlation between the plasma proteome quantified at baseline and three years later for each individual. This allowed us to classify the proteins into three groups: stable proteins, characterized by high temporal stability and low biological variability; signature proteins, with both high temporal stability and high biological variability; and unstable proteins, distinguished by low temporal stability regardless of biological variability (Figure 8 and Table S1). Functional enrichment analysis revealed that each of these protein groups was implicated in distinct biological processes. Thus, stable proteins participated in processes such as complement activation, blood coagulation, lipid transport (mainly apolipoproteins involved in high-density lipoprotein (HDL) assembly, cholesterol transport, and very-low-density lipoprotein (VLDL)-receptor binding), metal ion transport, and endopeptidase activity. Signature proteins were implicated primarily in the innate immune and inflammatory response and lipid transport (represented principally by HDL apolipoprotein components). Finally, unstable proteins were composed mostly of proteins involved in complement activation, principally through the classical pathway; serine proteases with endopeptidase activity; hemoglobins; blood coagulation proteins; and apolipoproteins (mainly VLDL apolipoprotein components) (Figure 8).

### 3.7. Performance of the On-Plate Sample Preparation Method 

Our original MS-based proteomics workflow relies on individual centrifugal devices for on-filter single-sample digestion. As an alternative, we also developed a 96-well plate format for reducing sample preparation time and improving the reproducibility of such procedures as protein digestion. This on-plate protocol was used to process 350 plasma samples from the AWHS cohort [35,36].

The examination of protein quantification accuracy based on the internal standard sample included in every TMT experiment and the biological samples, as described above, (Section 3.3) revealed that quantification was accurate in both cases (Figure S1A,B). Technical and biological variability were also assessed by estimating the variance at the scan, peptide, and protein levels. The technical and biological variances were similar to those found with the original protocol (Figure S1C) [22]. On-plate performance of the workflow was also evaluated before and after peptide-level fractionation. On average, 1845/2937 peptides corresponding to 390/774 proteins were quantitated before/after fractionation.

The analytical variability was evaluated by plotting the CV of the 1000 most abundant proteins quantified across 14 technical replicates against their ranked abundance (Figure S2A). We observed that ca. 99% of the proteins (988) had CV < 20, with only 12 proteins showing CV > 20 (Figure S2B). Interestingly, none of the 49 FDA-approved biomarkers quantified with our workflow showed CV > 20 (Figure S2C). Inter-individual (biological) variability was analyzed by plotting the CV of the 1000 most abundant proteins across 350 individuals that were analyzed via LC-MS/MS over two months against their ranked abundance (Figure S2D). Forty percent of proteins (403) showed CV < 20, and twenty-eight percent of proteins showed CV between 10 and 20 (Figure S2E). Regarding the FDA-approved biomarkers, only 15 out of 49 had CV > 20 (Figure S2F). These results revealed that the plate protocol had a similar performance to the original one and was therefore suitable for large-scale clinical studies.

## 4. Discussion

MS-based proteomics is increasingly entering regulated clinical and diagnostic settings [53,54,55], with the potential to yield predictive biomarker signatures that support clinical decisions, as well as to enable the prediction of patient evolution, provided that datasets of appropriate depth and size are available [56]. However, while routine clinical applications demand accuracy, reproducibility, and robustness, with low cost and high throughput to facilitate comparison within and between laboratories [57], MS-based analysis of human plasma remains challenging mainly because of both the large dynamic range in abundance of blood proteins and the presence of a few large, extremely abundant species that hamper the detection of lower abundance species [58]. Plasma depletion from highly abundant species and peptide-level fractionation have been used to circumvent these difficulties; nevertheless, depletion can introduce analytical bias due to the removal of lower abundance proteins either carried over by the depleted proteins or unspecifically bound to the antibodies used [14,15], and extensive fractionation is highly time-consuming [16]. This unfortunate trade-off between depth and throughput has led to a situation where only a few works on plasma proteomics have analyzed >500 samples with significant proteome coverage [17,18,19]. For these reasons, we developed an MS-based proteomics workflow aimed at the analysis of human plasma samples that benefits from accurate quantification and higher analysis throughput facilitated by multiplexed isobaric labeling, increased depth of detection attained via peptide-level fractionation, and robust statistical treatment by means of a validated, fully automated quantitation procedure [20,21,22,23,24,25]. Our workflow is characterized by (i) applicability to large sample cohorts, as illustrated by the analysis of >1300 human plasma samples, which can also be accomplished on 96-well plates; (ii) high-throughput capacity and satisfactory depth of detection, allowing the quantification of either 537 proteins at 29 samples per day (50 min per sample) without prior peptide-level fractionation or 917 proteins at 8 samples per day (180 min per sample) after fractionation; and (iii) high quantitative accuracy and reproducibility, with CV < 20% for 90% of the proteins quantitated (895 proteins).

Reproducibility issues in plasma depletion from high abundance proteins have long been known [59,60]. In this work, we demonstrated that this procedure induces changes in the plasma proteome that lead to biased peptide and protein quantification, as evidenced by the decreased intra-individual plasma proteome correlation upon depletion as compared to long-time correlation. Accordingly, this otherwise time-consuming, multistep procedure was not included in our workflow, which was evaluated for quantitative reproducibility based on the WSPP model to separately estimate scan-, peptide-, and protein-level variances to conclude that both technical and biological variances were within the expected values at the three levels. Quantitative accuracy was further supported by the very strong correlation found between the quantitative values provided by the workflow and biochemical measurements.

To investigate the sources of variability associated with our workflow, we first demonstrated both technical and biological reproducibility using five proteins with plasma concentration spanning five orders of magnitude (HAS, CP, FBLN1, PF4, and MMRN1). Then, analytical reproducibility (i.e., that related exclusively to the LC-MS/MS analysis) was shown with the 1000 most abundant species quantitated, where 90% of the proteins (895 proteins, including 67 of the 109 FDA-approved biomarkers) were found to have analytical CV < 20, the most common cut-off in diagnostic assays [52]. Finally, this 1000-protein set was also used to further assess biological reproducibility across 444 individuals, where 20% of the proteins (195 proteins, including 33 FDA-approved biomarkers) exhibited analytical CV < 20. The results support the capacity of our newly developed workflow to provide accurate protein quantification, with quantitative changes accounting for the inter-individual variability of plasma proteins instead of technical or analytical variability. The functional categories found enriched with the subset of highly biologically variable proteins, i.e., those quantitated across at least 80% of the subjects with CVt < 12 and CVb > 30, are in agreement with a previous report, where high-abundance erythrocyte-specific proteins (such as hemoglobin), acute-phase proteins (such as serum amyloid A1 protein, SAA1; and C-reactive protein, CRP), lipoproteins (such as apolipoprotein (a), LPA), and proteins from the blood coagulation system (such as fibrinogen, FG; platelet basic protein, PPBP; and platelet factor 4 variant, PF4V1) were described as the species with the highest biological variability [45]. Likewise, the selection of a longitudinal prospective cohort comprising baseline and 3-year follow-up visit samples enabled us to examine the long-term temporal stability of the plasma proteome. The majority of individuals (84%) and proteins (94%) showed significant correlation between these two time points, suggesting that the plasma proteome is highly stable in time, with much higher inter- than intra-individual variability. Similar observations have been reported previously but were either based on a few selected proteins, using smaller longitudinal cohorts, or over shorter periods of time [46,47,48,49,50,51].

According to their behavior over time and their biological variability, plasma proteins were divided into three different groups: stable proteins (high temporal stability with low biological variability), signature proteins (high temporal stability and biological variability), and unstable proteins (low temporal stability with either low or high biological variability). Stable proteins comprise the second largest group, with species involved in such processes as complement activation, endopeptidase activity, blood coagulation, and lipid transport (mainly apolipoproteins involved in HDL assembly and VLDL-receptor binding). The largest subgroup of stable proteins is composed of complement factors, which are involved in the pathogenesis of many inflammatory diseases; thus, C3 and C4 circulating levels are used to monitor patients with systemic lupus erythematosus [61]. The low biological variability of these complement components is accounted for by the fact that hereditary complement deficiencies are rare and generally associated with recurrent bacterial infections [62]; however, a reduced group of complement factors were classified as unstable proteins due to their high biological variability and low temporal stability. Moreover, some studies reported low biological variability for fibrinogen, clotting factors, and antithrombin, while fibrinolytic molecules, such as plasminogen activator inhibitor 1 and fibrinopeptide A, were found highly variable [63].

Signature proteins, which also show low temporal variability, but, unlike stable proteins, are highly biologically variable, are implicated in immune and inflammatory responses and in lipid transport (represented mainly by HDL apolipoprotein components). LPA plasma levels, which have long been associated with increased risk of CVD, have previously been shown to vary up to >1000 times across individuals of the same population [64] as a consequence of its high genetic variability and the involvement of other genes related to its synthesis and metabolism. In contrast, LPA plasma concentrations are not altered by environmental factors and are thought to be relatively constant throughout a person’s lifetime [65]. Moreover, plasma lipid levels and risk of CVD are highly heritable, with estimates ranging from 30% to 60% [66,67,68], and genetic polymorphisms in apolipoprotein-encoding genes constitute important modulators of serum lipid profiles and CVD susceptibility. Human studies have identified polymorphisms in the APOA4 gene that associate with its plasma levels, inter-individual variability in cholesterol levels, and risk of coronary heart disease [69]. Moreover, circulating HP levels have previously been demonstrated to be markedly reproducible in healthy individuals over a 4-month period [70]. It has also been previously described that the intra-individual variations (with CVs ranging from 5% to 52%) of some serum immunoglobulins, such as IgG, IgA, and IgM, were smaller than inter-individual variations (with CVs ranging from 15% to 108%) [71].

These findings indicate that both stable and signature proteins may serve as reliable biomarkers of different nature. Thus, alterations to the former reflect an individual’s deviation from baseline population health, whereas signature proteins might constitute a personalized fingerprint of disease (e.g., by reflecting immune-inflammatory pathways associated with metabolic health). In contrast, unstable proteins (the largest group, composed mainly of complement and blood coagulation components, serpins with endopeptidase activity, hemoglobins, and VLDL apolipoprotein components) have low diagnostic or prognostic capacity due to their high temporal and biological variability. Thus, high-abundance erythrocyte-specific proteins, and specifically hemoglobin subunits alpha (HBA2), beta (HBB), and delta (HBD), often show high variability due to a different extent of erythrocyte hemolysis or platelet contamination during plasma preparation and harvesting [57].

The implementation of personalized medicine in the analysis of otherwise healthy individuals for estimation of disease risk and medical interpretation is not yet clear. High-prevalence diseases like CVD involve many distinct genes and biological pathways, together with environmental contributors that have not been fully established yet [72], and detailed molecular analysis of blood samples will help predict, diagnose, and treat diseases [73]. The workflow presented here generated a rich proteomics dataset on two visits (baseline and 3-year follow-up) of 444 individuals that provided insight into potential personal CVD markers and patterns. Inter-individual differences, ultimately reflected in the signature plasma protein group, are likely due to genetic variability; however, environmental contributors cannot be ruled out, suggesting that these potential markers are actionable through lifestyle changes.

Finally, the proteomics workflow put forward in this study should be easily adaptable to other body fluids, such as cerebrospinal fluid, urine, or saliva, all of which are tantalizing sources of disease biomarkers.

## Data Availability

MS raw data have been deposited in Peptide Atlas (http://www.peptideatlas.org/PASS/PASS01382 and http://www.peptideatlas.org/PASS/PASS01522, accessed on 17 August 2024.

## Supplementary Materials

The following supporting information can be downloaded at: https://www.mdpi.com/article/10.3390/biomedicines12092118/s1, Figure S1: On-plate protein quantification accuracy of the large-scale proteomics workflow; Figure S2: On-plate analytical and biological variability of the large-scale proteomics workflow; Table S1: Classification of plasma proteins according to their temporal stability and biological variability.
