# Supplementary material for: A Multiplexed Quantitative Proteomics Approach to the Human Plasma Protein Signature
Source: Biomedicines. 2024 Sep 18;12(9):2118. doi: 10.3390/biomedicines12092118 (PMC11428418; doi:10.3390/biomedicines12092118)
Supplement: Supplementary file 1 [file biomedicines-12-02118-s001.zip › Figure S1.pptx]

## Slide 1
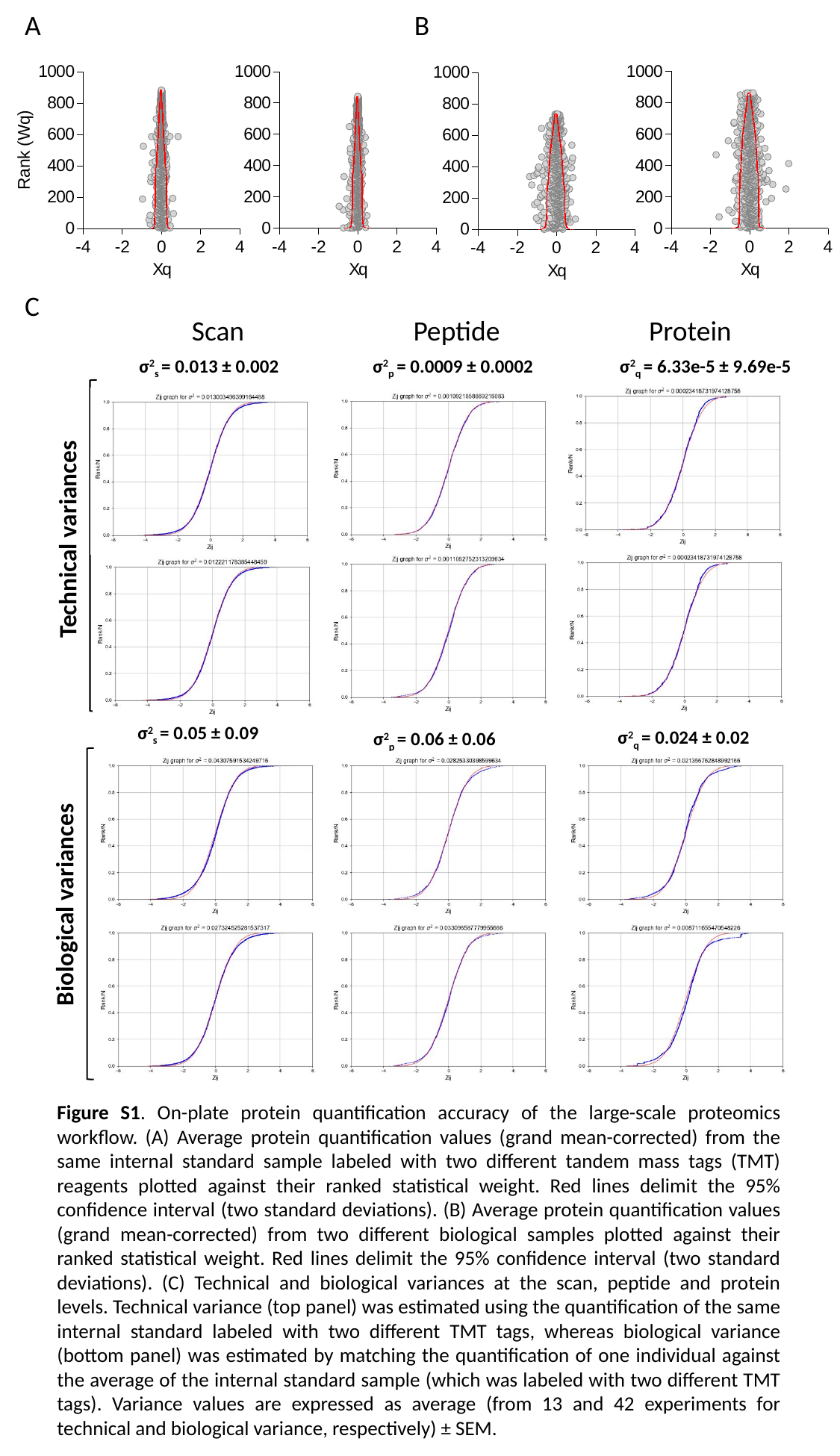

A
B
C
Scan
Peptide
Protein
σ2s = 0.013 ± 0.002
σ2p = 0.0009 ± 0.0002
σ2q = 6.33e-5 ± 9.69e-5
Technical variances
σ2s = 0.05 ± 0.09
σ2q = 0.024 ± 0.02
σ2p = 0.06 ± 0.06
Biological variances
Figure S1. On-plate protein quantification accuracy of the large-scale proteomics workflow. (A) Average protein quantification values (grand mean-corrected) from the same internal standard sample labeled with two different tandem mass tags (TMT) reagents plotted against their ranked statistical weight. Red lines delimit the 95% confidence interval (two standard deviations). (B) Average protein quantification values (grand mean-corrected) from two different biological samples plotted against their ranked statistical weight. Red lines delimit the 95% confidence interval (two standard deviations). (C) Technical and biological variances at the scan, peptide and protein levels. Technical variance (top panel) was estimated using the quantification of the same internal standard labeled with two different TMT tags, whereas biological variance (bottom panel) was estimated by matching the quantification of one individual against the average of the internal standard sample (which was labeled with two different TMT tags). Variance values are expressed as average (from 13 and 42 experiments for technical and biological variance, respectively) ± SEM.
