# Supplementary material for: A Multiplexed Quantitative Proteomics Approach to the Human Plasma Protein Signature
Source: Biomedicines. 2024 Sep 18;12(9):2118. doi: 10.3390/biomedicines12092118 (PMC11428418; doi:10.3390/biomedicines12092118)
Supplement: Supplementary file 1 [file biomedicines-12-02118-s001.zip › Figure S2.pptx]

## Slide 1
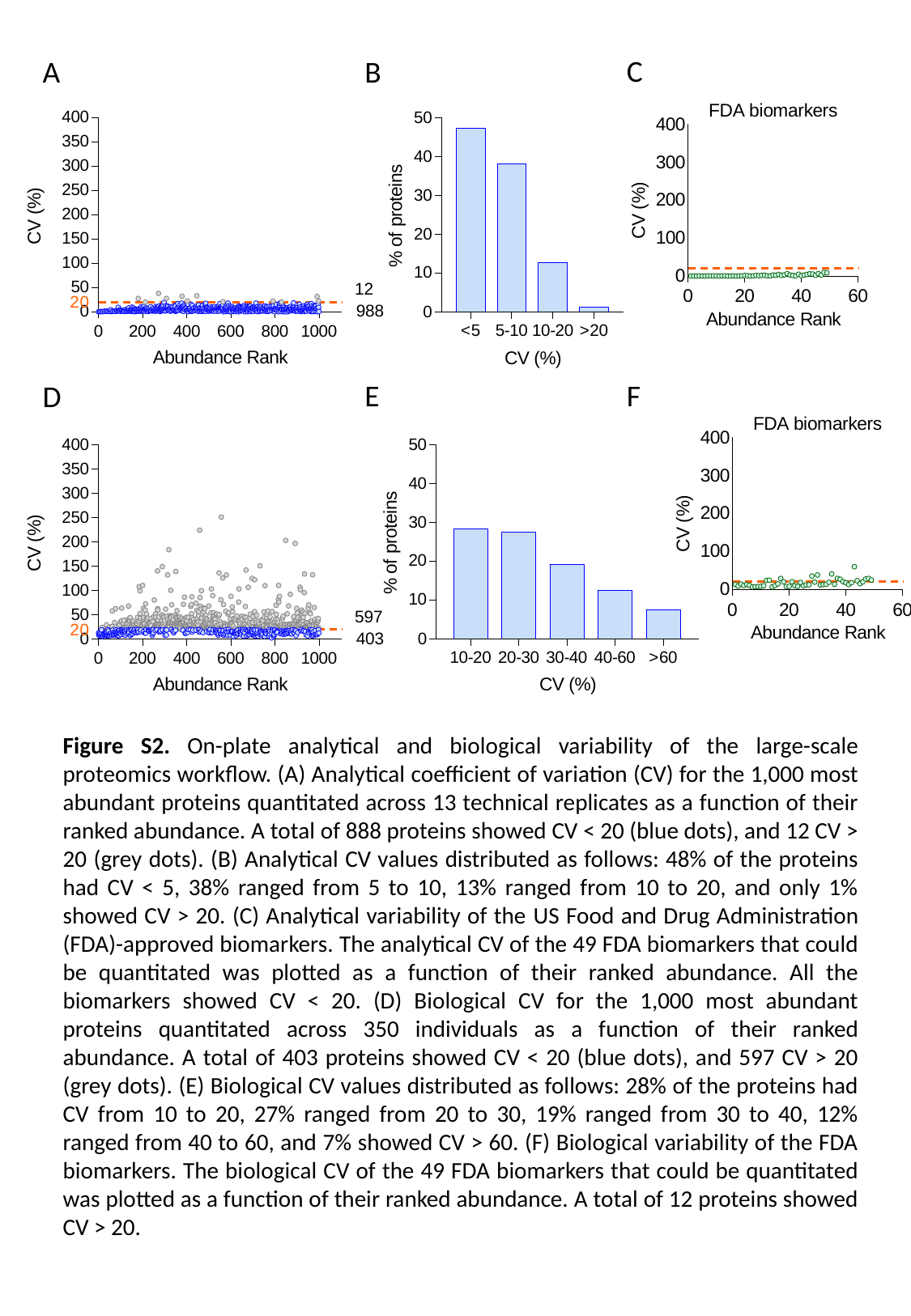

C
B
A
12
988
F
E
D
597
403
Figure S2. On-plate analytical and biological variability of the large-scale proteomics workflow. (A) Analytical coefficient of variation (CV) for the 1,000 most abundant proteins quantitated across 13 technical replicates as a function of their ranked abundance. A total of 888 proteins showed CV < 20 (blue dots), and 12 CV > 20 (grey dots). (B) Analytical CV values distributed as follows: 48% of the proteins had CV < 5, 38% ranged from 5 to 10, 13% ranged from 10 to 20, and only 1% showed CV > 20. (C) Analytical variability of the US Food and Drug Administration (FDA)-approved biomarkers. The analytical CV of the 49 FDA biomarkers that could be quantitated was plotted as a function of their ranked abundance. All the biomarkers showed CV < 20. (D) Biological CV for the 1,000 most abundant proteins quantitated across 350 individuals as a function of their ranked abundance. A total of 403 proteins showed CV < 20 (blue dots), and 597 CV > 20 (grey dots). (E) Biological CV values distributed as follows: 28% of the proteins had CV from 10 to 20, 27% ranged from 20 to 30, 19% ranged from 30 to 40, 12% ranged from 40 to 60, and 7% showed CV > 60. (F) Biological variability of the FDA biomarkers. The biological CV of the 49 FDA biomarkers that could be quantitated was plotted as a function of their ranked abundance. A total of 12 proteins showed CV > 20.
